# Using Discrete Choice Experiments (DCEs) to Compare Social and Personal Preferences for Health and Well-Being Outcomes

**DOI:** 10.1177/0272989X251378427

**Published:** 2025-10-16

**Authors:** Nyantara Wickramasekera, An Thu Ta, Becky Field, Aki Tsuchiya

**Affiliations:** Sheffield Centre for Health and Related Research, University of Sheffield, Sheffield, UK; Sheffield Centre for Health and Related Research, University of Sheffield, Sheffield, UK; Sheffield Centre for Health and Related Research, University of Sheffield, Sheffield, UK; Sheffield Centre for Health and Related Research, University of Sheffield, Sheffield, UK; School of Economics, University of Sheffield, Sheffield, UK

**Keywords:** social and personal preferences, resource allocation, discrete choice experiment, health and well-being outcomes

## Abstract

**Background:**

Economic evaluations in health typically assume a nonwelfarist framework, arguably better served by preferences elicited from a social perspective than a personal one. However, most health state valuation studies elicit personal preferences, leading to a methodological inconsistency. No studies have directly compared social and personal preferences for outcomes using otherwise identical scenarios, leaving their empirical relationship unclear.

**Aim:**

This unique study examines whether the choice of eliciting preferences from a social or personal perspective influences valuations of health and well-being outcomes.

**Methods:**

Using discrete choice experiments, social and personal preferences for health and well-being attributes were elicited from the UK general public recruited from an internet panel (*n* = 1,020 personal, *n* = 3,009 social surveys). Mixed logit models were estimated, and willingness-to-pay (WTP) values for each attribute were calculated to compare differences between the 2 perspectives.

**Results:**

While no significant differences were observed in the effects of physical and mental health, loneliness, and neighborhood safety across the 2 perspectives, significant differences emerged in WTP values for employment and housing quality. For instance, other things being the same, personal preferences rate being retired as more preferable than being an informal caregiver, but the social preferences rate them in the reverse order.

**Conclusion:**

Our findings demonstrate that the perspective matters, particularly for valuing outcomes such as employment and housing. These findings indicate that the exclusive use of personal preferences to value states such as employment and housing quality may potentially lead to suboptimal resource allocation, given that such valuations reflect individual rather than societal benefit. This highlights the importance of considering perspective especially in the resource allocation of public health interventions.

**Highlights:**

- Personal preferences were not aligned with social preferences for employment and housing quality outcomes.
- Respondents valued health outcomes the same in both social and personal perspectives.
- Using personal preferences in public health resource allocation decisions may not reflect societal priorities.

In the health care, social care, and public health sectors, the market mechanism cannot be expected to work efficiently, because the existence of uncertainty and asymmetry of information result in market failure.^1^ As a result, many countries have systems to fund these services from public resources, which require a nonmarket mechanism to set budget allocations. One established way to allocate public resources efficiently is to subject different policy options to economic evaluation that contrasts the value of inputs (resource use) and outputs (health and wellbeing outcomes).^2^

Economic evaluation frameworks such as those set out by the National Institute for Health and Clinical Excellence (NICE) implicitly assume that social welfare is maximized through the maximization of population health measured in terms of the quality-adjusted life-year (QALY), where health state values are captured through average population preferences.^3^ In other words, the approach does not exploit variation in preferences across people, which is at odds with welfarism. Welfarism assumes that social welfare increases in individual utility over outcome states, as judged by the individuals themselves.^4^ People are expected to have different willingness (and ability) to pay for the same services, and allocating public resources based on 1 set of preferences would be inefficient. For example, if a service is made available because the average preference for it clears an exogenously set threshold, those who do not value it as much may still access this; or if the service is withheld because the average preference for it does not clear the threshold, those who value it much higher would be unable to access it. The approach, instead, can be interpreted as nonwelfarist and is better aligned with a central tenet of the NHS: equal access for equal needs regardless of ability to pay.^5^

On the other hand, health state valuation exercises that elicit the relative tradeoff between quality of life and quantity of life that operationalize the QALY, typically use a personal preference. Health and well-being outcomes can be valued using personal, social, or other preferences.^6,7^ A personal preference valuation would ask respondents, for example, whether they would choose for themselves to live a longer time in poorer health or the other way round, whereas a social preference valuation would ask respondents whether, if it was their job to choose on behalf of society, they would choose for fellow citizens to live longer lives in poorer health or the other way round. Arguably, valuation from a personal preference is better aligned with a welfarist approach to economic evaluation while valuation from a social preference is better aligned with a nonwelfarist approach.^8,9^

Separately from the normative issue of whether nonwelfarist economic evaluation would be better served by outcome valuation from a social perspective, of interest is whether or not the perspective affects the preferences elicited. However, while a few studies have compared the personal perspective with other (proxy, nonuse, and socially inclusive personal) preferences, we are not aware of any that compared the social and personal preferences for health and well-being outcomes using otherwise (near) identical valuation scenarios, and as a result, little is known about their empirical relationship.

In child health valuation, a study used scenarios in which respondents chose for themselves, on behalf of both another adult and a child.^10^ It found larger differences between proxy preferences for another adult and for a child compared with between personal preferences and proxy preferences for another adult.

In ecological economics, the distinction between “citizen” and “consumer” perspectives has been explored.^11^ Empirical studies focused on forest preservation^12^ and rural landscape preservation,^13^ comparing social and personal willingness-to-pay (WTP) amounts, yielding mixed results. However, it is important to note that in these studies, social WTP refers to the amount individuals consider paying (e.g., through taxation) as citizens, reflecting a socially inclusive personal or nonuse perspective, rather than a purely social perspective.

Our study investigates whether the choice of social or personal perspective affects people’s preferences over health and well-being outcomes. If the perspective proves to have no significant effect on the elicited preferences, then the normative debate on which perspective to use would be a moot point. If, furthermore, one preference proves more difficult to obtain than the other, then the easier one to elicit could serve as a proxy for the other. We measure how people trade off across dimensions of health and well-being from both perspectives, using (near) identical valuation exercises. An online sample was recruited to survey members of the UK public. WTP for the well-being attributes was compared to identify any differences between social and personal preferences.

## Methods

### Attributes and Levels of the Discrete Choice Experiment: SIPHER-7

The discrete choice experiment (DCE) attributes are based on SIPHER-7, a suite of 7 well-being indicators,^14,15^ developed as a part of the Systems-science in Public Health and Health Economics Research (SIPHER) project^16^ to depict well-being outcomes in economic evaluations of public health policies across government sectors. The 7 indicators of SIPHER-7 include 2 ordered categorical attributes in health (effects of physical health and effects of mental health), 3 ordered categorical attributes in well-being (loneliness, housing quality, and neighborhood safety), 1 nonordered attribute on employment, and a continuous attribute on household finances (Table 1). Each SIPHER-7 attribute is linked directly or indirectly to questions in the UK Household Longitudinal Study^17^ so that the UKHLS sample can be allocated to SIPHER-7 profiles.

**Table 1:** Attribute and Level Descriptions of the Social DCE^a^

Attributes of the Social DCE	Levels of the Social DCE
**Effect of physical health (P):** This is about how a person’s physical health affects their activities.	None of the time (reference level); Little of the time; Some of the time; Most of the time; All of the time
**Effect of emotional problems (M):** This is about how a person’s emotional problems affect their activities.	None of the time (reference level); Little of the time; Some of the time; Most of the time; All of the time
**Loneliness (L):** This is about whether people feel lonely for whatever reason and left out from others.	Hardly ever (reference level); Some of the time; Often
**Household spending money (S):** This is the amount of money that a household has each month after their tax, national insurance, pension contributions, and their housing costs (e.g., rent, mortgage payments, etc.) have been paid. It is the amount of spending money that the whole household has left to spend each month, including on bills, groceries, and leisure activities.	£690/mo [that is, £170/wk] £1,040/mo [that is, £260/wk] £1,380/mo [that is, £340/wk] £1,730/mo [that is, £430/wk] £2,080/mo [that is, £520/wk]
**Employment (E):** This is about people’s main daily activity.	Full-time employment (this includes self-employment and being on leave [reference level]) Part-time employment (this includes self-employment and being on leave) Job-seeking (unemployed and looking for employment) Full-time education/training/apprenticeship Taking care of a family member with chronic illness or disability Not working, and not looking for paid employment (for example retired, looking after the family/home, volunteering, etc.)
**Quality of housing (H):** This is about whether somebody’s home 1) is in a good state of repair, 2) has reasonable facilities for cooking and washing, and 3) provides reasonable warmth when it is cold outside.	Good (reference level); Fair; Poor
**Neighborhood safety (N):** This is about the area people live in and how safe they feel within their immediate neighborhood	Hardly ever (reference level); Some of the time; All of the time

DCE, discrete choice experiment.

a The attributes wording for the personal survey were adapted, for example, from “their” health to “your” health. The levels were identical in both surveys.

### The Original DCE Study

An earlier DCE study elicited the preferences of the general public for the SIPHER-7 indicators from a personal perspective.^18^ This DCE design was based on a D-efficient, unlabeled, partial profiles design generated using Ngene, for a model including interactions between employment and household finances, with nonzero priors obtained from a pilot study (*n* = 100). The partial profiles design had at least 2 of the 6 categorical attributes tied to simplify the choice tasks. No opt-out option was provided. A total of 120 choice tasks were split into 12 blocks, each containing 10 DCE tasks. This design was adapted for the social and personal DCEs in the current study.

### Developing the Social and the Personal Versions of the DCE

In the current study, the instructions from the original DCE were adapted for the social preference DCE by introducing an imaginary population. While social preference choice tasks can be set across 2 groups (e.g., “which group is better off?”), our social preference choice tasks involved 2 alternative outcomes for a single group. This approach focused on eliciting the relative values of the attributes and levels of SIPHER-7 as a measure of efficiency, avoiding interpersonal comparisons of well-being that may conflate efficiency (which alternative is “better”) with equity (which alternative is “fairer”) from choosing between 2 groups. In addition, although the social perspective DCE could involve 2 groups (“you,” the respondent, are not in either group), the personal perspective DCE can only involve 1 party (“you,” the respondent), and the 2 DCEs should be as similar as possible to each other. The instructions specified that everybody in this imaginary population lived in 3-person households. The household size of 3 was strategically chosen to ensure the realism of specific scenarios (e.g., unemployment with high spending requiring multiple adults; or a single-person household would have rendered the informal caregiving attribute level inapplicable), and it establishes a relatable household size for the social preference task, approximating the average UK household size.

The draft social perspective DCE underwent cognitive interviews^19,20^ with 6 general public participants (3 men, 3 women; 2 aged 18–34 y, 2 aged 35–54 y, 2 aged 55+ y; non–university educated). These interviews were conducted online by a social research agency, using the think-aloud method. Participants were probed on their experience with the introduction and instructions, their approaches to answering the choice tasks, whether they were able to think in terms of social preferences, and their understanding of the language used, and they were invited to provide suggestions for improvement.

In the introduction, respondents were prompted to envision advising policy makers on public policy, with explanations provided about policy makers’ roles and examples of how different policies might affect people differently across the well-being dimensions (e.g., “a policy might help people get a better-paid job but worsen their stress levels”). Respondents were asked to think about what good lives and good societies look like. Each choice task instructed respondents to indicate the following: “I think policy makers should try to bring about: Life situation A” (or Life situation B). There was no indifference option offered.

**Figure 1:**
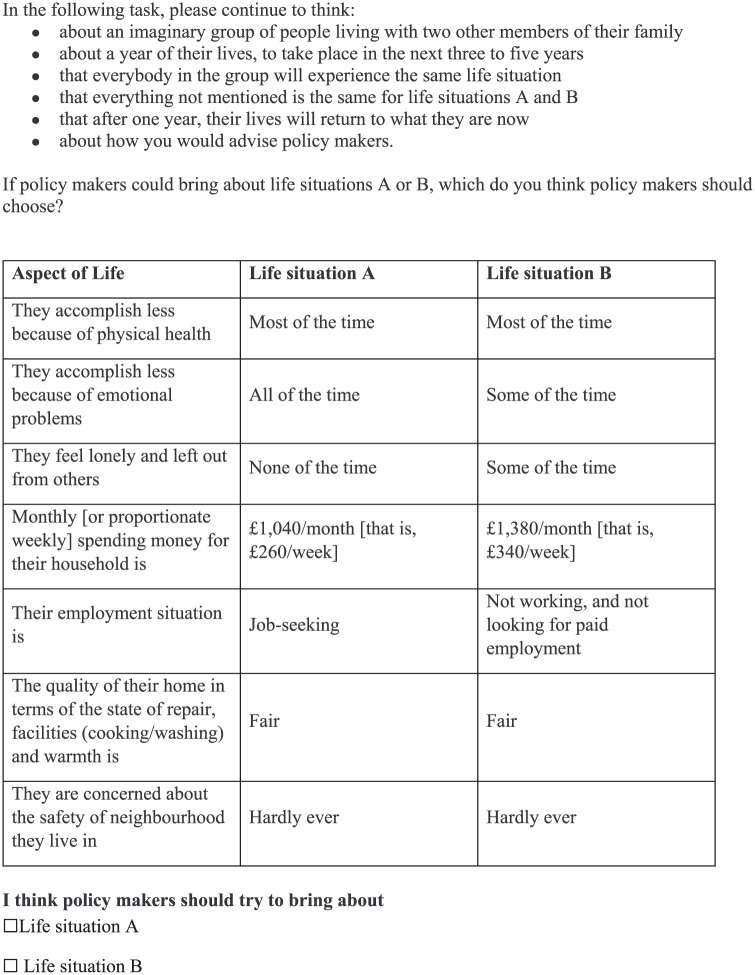
Discrete choice experiment social preference task.

Overall, participants appeared to understand most of the instructions and were able to explain how they weighed up the different choices presented. However, it was unclear whether these 6 participants answered the social preference DCE based on their personal preferences and experiences or whether their social and personal preferences aligned. Some referred to their personal situations (e.g., being retired, their own ill health experiences, etc.), prompting adjustments to remind respondents of the social perspective in the survey instruction and those preceding each DCE: “We would like you to think about a group of people living in the UK now. . . . If policy makers could make their lives look like A or like B, which do you think policy makers should try to bring about?” Respondents were also reminded that “You may have a personal preference between the two life situations, but we are interested in something a bit different.” Moreover, the introduction was simplified, and key terms were clarified based on feedback from participants. For instance, “disposable income” was replaced with “spending money” or housing being in a “reasonable” state of repair was replaced with “good” for better understanding. An example of the final version of the social perspective DCE task is presented in Figure 1.

**Figure 2:**
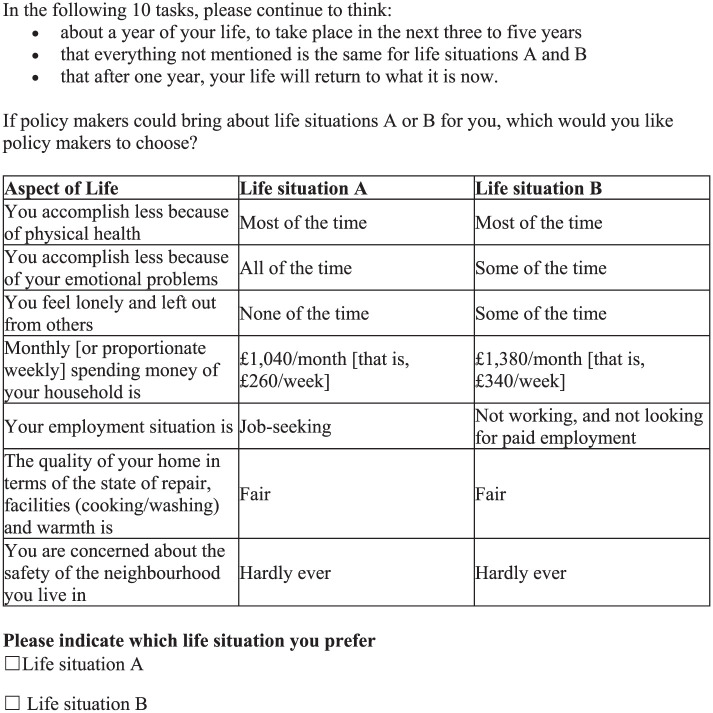
Discrete choice experiment personal preference task.

Once the social perspective DCE was finalized, the personal perspective DCE was developed by maintaining the wording from the social version and adjusting it to the personal perspective where necessary. The DCE attributes were adapted, such as changing “their” health to “your” health. Household size was removed from the instructions, while the same levels of household spending money were used for all respondents answering the personal DCE (household spending was equivalized by each respondent’s household size in the analysis; see below). An example of the final version of the personal perspective DCE task is presented in Figure 2.

### The Surveys

Both DCE surveys were developed on Qualtrics with identical structures. In the first section, participants consented to take part and completed age and gender questions. The second section contained the DCE tasks. A series of screens displayed background information about the SIPHER-7 indicators and provided details of the decision-making context. Participants completed 3 practice tasks of increasing difficulty: the first 2 had a logically better alternative (e.g., dominance check), whereas the third did not. After the practice tasks, participants were randomized into 1 of 12 blocks and completed 10 DCE tasks. The debriefing questions followed, which contained a free-text comment box and multiple response questions with a list of positively and negatively worded feedback statements in which respondents were asked to select all the statements that applied to them. In the final section of the survey, participants completed standard sociodemographic questions and the SIPHER-7 suite.

### Sampling and Recruitment

Respondents to both surveys were recruited in parallel from a single commercial internet panel (Pureprofile). The target sample size was 3,000 for the social perspective DCE and 1,000 for the personal. The inclusion criteria were participants aged 18 years or older and living in the United Kingdom. To ensure a sample representative of the UK public, stratified sampling was used with age band and gender quotas. To be included in the study, respondents also needed to meet a minimum time to completion of 5 min: the same cutoff was used in the original personal DCE,^18^ which corresponds to 30% of the average time taken by the respondents of that survey. Other data quality controls included preventing the same person from entering multiple submissions, bot detection using Captcha questions, and having a valid commercial panel ID.

### DCE Data Analysis

We analyzed the data using a mixed logit model to account for repeated observations from each respondent (within the survey) and individual-level preference heterogeneity. Random parameters with a normal distribution for all attributes other than spending money were used. The model was estimated with 1,000 scrambled Halton draws using the mixlogit command in Stata.^21^ Additional models were estimated using Bayesian estimation using the garbage_mixl command in Stata,^22^ with results presented in the supplementary materials.

The DCE data are modeled as follows:

(1) $$\begin{array}{c} U=\beta_{1}P+\beta_{2}M+\beta_{3}L+\beta_{4}\ln\left(S'\right)\\ +\beta_{5}E+\beta_{6}H+\beta_{7}N+\varepsilon \end{array}$$

where U is the value of SIPHER-7 profiles; the β s are vectors of the distribution of coefficients for each of the categorical attributes ( P to N of SIPHER-7), with the exception of $\beta_{4}$ (spending money) modeled as a fixed linear coefficient. The best level was used as the reference for all ordered attributes, except for employment, which was arbitrarily defined as full-time employment (see further details in Table 1). Based on the OECD square root scale method,^23,24^ spending money (S′) was equivalized for each respondent using the assumed household size of 3 ( $S/\sqrt{\mathrm{household}\;\mathrm{size}})$ for the social DCE (where respondents were asked to think about an imaginary group of 3 people) and using respondents’ own household size for the personal DCE, which was then modeled as the natural logarithm (ln(S′)) to allow for nonconstant marginal value of money.

The DCE design allowed for interactions between household spending money and employment. The Bayesian information criterion using mixed logit showed that including interaction effects of spending money and employment attributes resulted in a worse fit in both personal and societal perspective data. Therefore, we opted to estimate a main effects model for both perspectives.

To facilitate the comparison between the 2 DCE surveys, preferences were standardized in terms of WTP. Marginal rates of substitution given by the WTP to improve each of the nonmonetary attributes to their best level were calculated. That is the amount of spending money that respondents were willing to trade off in exchange for achieving the most preferred level in another well-being attribute. Confidence intervals for the WTP were calculated using the delta method.^25^

The WTP amounts for a change, for example, from the different employment statuses (*E*) to the reference category (full time employment) are calculated as

$$\mathrm{WT}P_{E}=\frac{\partial U/\partial E}{\partial U/\partial S}=\left(\frac{\beta_{5}}{\beta_{4}}\right)S(2)$$

All data analyses were carried out in Stata v17.

### Sensitivity Analyses

The WTP for each attribute level was recalculated by excluding those who selected “not sure about my responses” from the feedback statements and separately by excluding those who failed at least 1 of the 2 practice tasks with a logically determined answer.

### Respondent Feedback

Qualitative feedback obtained from the free-text comments for the 2 surveys was imported into Excel and “cleaned” to remove comments that were illegible or not insightful. One researcher (B.F.) then read through all comments and coded them to enable grouping of similar comments into categories. Codes from the 2 DCE surveys were then compared for similarities and differences, and the main categories were identified.

Quantitative feedback generated from the list of debriefing statements was analyzed using descriptive statistics, and the frequencies of each statement were compared across the 2 DCEs.

## Results

### Participant Characteristics

The online survey took place between March and April 2022. The social preference DCE included 3,009 participants, after removing 515 respondents for speeding (submitting the survey in less than 5 min) and removing 1,233 (26%) respondents who dropped out of the survey. The personal preference DCE included 1,020 in the analysis, after removing 126 for speeding and another 288 (20%) people who dropped out. Across both surveys, the dropout rates were not significantly different, and speeders were not different from the included sample in terms of gender, but they were more likely to be younger than 45 years.

In total, 4,029 respondents were included in the main analysis. These participants completing the social and personal preference DCEs were broadly similar across sociodemographic characteristics and SIPHER-7 except in employment status, having children, and warmth of their house (Supplementary Tables 1 and 2). The sample is also representative of the UK general population in terms of gender and age. Males and females were equally represented in the samples, with representation of all age groups, ethnicities, and education levels. The median time taken to complete the 2 surveys was significantly different, with 11.3 min for the social and 10.5 min for the personal surveys.

### Modeled Preferences

The modeled regression coefficients for the social and personal DCEs were as expected, with all coefficients for the ordered variables negative when less preferred attribute levels were compared with the reference levels; the spending money coefficient was positive, indicating that more money is preferred. The most preferred employment category was full-time employment. All coefficients were statistically significant at the 5% level except part-time employment in the personal and social preference DCEs and emotional problems affecting little of the time in the social preference DCE (Supplementary Table 3). The overlapping confidence intervals for the lower levels of physical health and emotional problems parameters suggest that respondents might have grouped “a little of the time” and “some of the time” together. The standard deviation of most of the random coefficients were significant, indicating strong preference heterogeneity (Supplementary Table 3).

**Figure 3:**
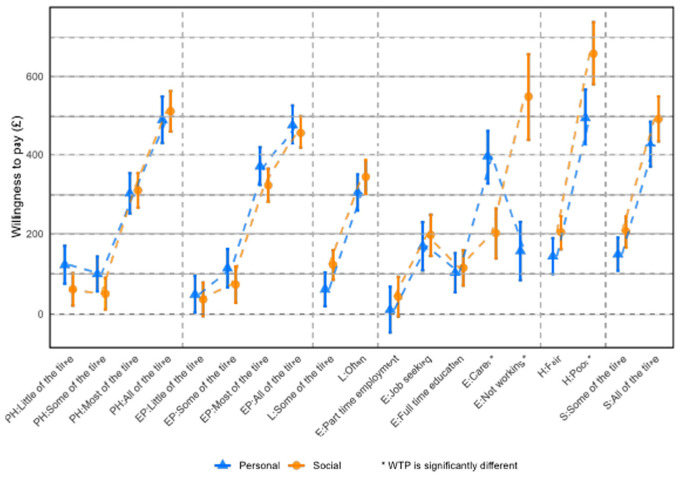
Willingness to pay to avoid a given level by attribute.

There was no logical ordering for the employment categories. The social and personal perspectives agreed to rank full-time employment first, followed closely by part-time employment and full-time education (Figure 3). Social preferences followed these by job seeking, taking care of a family member with chronic illness or disability (or, hereafter, “informal caregiving”), and not working and not looking for paid work (or “not working”) the worst. Personal preferences had “not working,” followed very closely by “job seeking,” and finally “informal caregiving.”

The relative ordering of the worst levels across the attributes for social and personal preferences was different (Figure 3 and Supplementary Table 3). For social preferences, the worst ranked attribute was housing quality, followed by not working, effect of physical health, neighborhood safety, effect of emotional problems, and being lonely. For personal preferences, the worst ranked attribute was the effect of physical health, followed closely by housing quality, effects of emotional problems, neighborhood safety, informal caregiving, and being lonely.

When comparing WTP amounts for different attribute levels, we observed similar patterns between social and personal preferences, with the exception of employment attribute levels related to informal caregiving, not working, and poor quality of housing (Figure 3 and Supplementary Table 3). From the social preference perspective, not working was seen as the worst employment option; this has the biggest gap in preferences between adjacent levels when comparing social and personal preferences.

### Internal Validity, Qualitative Feedback, and Sensitivity Analyses

In both practice tasks 1 and 2, most participants (>78%) passed the dominance test and selected the alternative that is logically better (Supplementary Table 4). Overall, the feedback indicated that participants’ experience of completing the DCE was mostly positive (Supplementary Table 4). For instance, 45.94% of social survey participants and 47.65% of personal survey participants felt the tasks being asked were clear, while 56.78% and 54.31%, respectively, found the survey interesting. Across the 2 surveys, the respondents in the social preference survey were significantly more likely to be unsure about their answers (S: 14.76% v. P: 8.92%), while the respondents in the personal preference DCE were significantly more likely to state that they were confident about their answers (S: 32.81% v. P: 37.35%). In addition, significantly more respondents in the personal preference DCE said that “the life situations were unrealistic” (S: 10.47% v. P: 14.12%) and “the tasks were boring” (S: 3.82% v. P: 5.49%) compared with those in the social preference version.

Qualitative feedback was given by 244 (8.1%) and 63 (6.2%) respondents in the social and personal surveys, respectively, after removing illegible or not insightful comments (including 48 [4.7%] from the personal perspective and 147 [4.9%] from the social perspective, which consisted of remarks such as “thank you” or “thought provoking”). A number of them (S: 61 [2%]; P: 32 [3.1%]) commented on what the respondents focused on most when making their choices, and of these, 17 (27.9%) and 9 (28.1%) referred to household finances. Despite the DCE label of spending money, “income” was a term that was often used by these respondents. After household finances, the effects of physical and emotional health and employment were mentioned. Respondents from both surveys mentioned difficulty of answering (S: 32 [1.1%]; P: 8 [0.8%]) about the general challenge of weighing up competing aspects and the scenarios being unrealistic (S: 7 [0.2%]; P: 2 [0.2%]). Comments about the scenarios not being personally relevant were made by 5 (0.5%) respondents of the personal survey (e.g., because they are retired and do not plan to go back to work or education) but none from the social survey.

**Figure 4:**
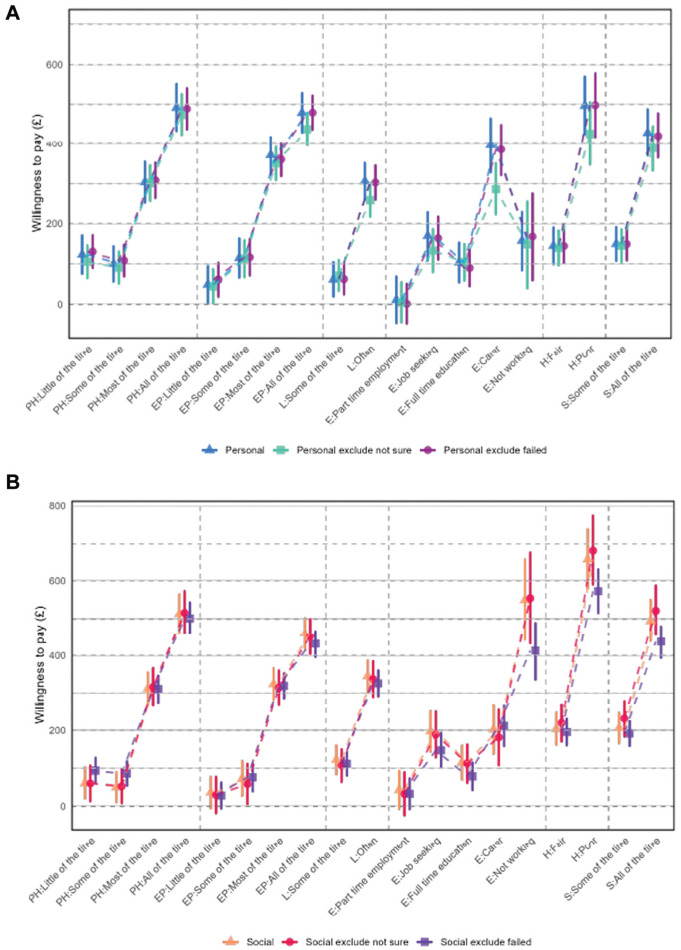
(a) Willingness to pay (WTP) for attribute levels for main effects and sensitivity analyses personal preferences. (b) WTP for attribute levels for main effects and sensitivity analyses social preferences.

Sensitivity analysis 1 was carried out excluding 13% of respondents, who said they were not sure about their answers provided for the DCE task. Sensitivity analysis 2 excluded 29% of respondents, who failed a practice task. The results show that WTP differences between sensitivity analyses and the main analysis are generally small and not significant (Figure 4a and b).

## Discussion

While there are methodological debates about the perspectives to be used in the valuation of health and well-being states that relate to the normative debate on welfarism and nonwelfarism, not much is known about the actual difference that the choice between social and personal perspectives may have on outcome valuation.

This study conducted 2 online DCE surveys to value the relative importance of 7 well-being indicators (SIPHER-7) to compare social WTP and personal WTP to improve the nonmonetary attributes of a given SIPHER-7 profile to the best levels. The 2 surveys were designed to be as similar as possible to each other, and respondents were randomized between the two, so that any differences observed can be attributed to the different perspectives used.

The aspects that resulted in significant differences between the social and personal preferences are employment and housing quality, so that WTP to improve employment and housing quality from the worst to the best combinations can be significantly different from each other depending on the perspective. For example, the different rankings of the employment categories mean that if an informal caregiver retires, other things being the same, this would be an improvement from a personal perspective but a deterioration from a social perspective.

Another notable difference is the reversed ranking of the employment categories of informal caring and not working in the 2 perspectives. It is possible that the social perspective encouraged respondents to think about how useful people’s economic activities are to society and the economy and therefore regard not working as not contributing to the economy. This could have led them to view not working as less beneficial, making it the least desirable attribute level. In contrast, it is also possible that the personal perspective encouraged respondents to not only think about oneself providing informal care but also about a family member needing informal care.

Of the 2 DCE surveys, respondents appear to have found the social perspective DCE more difficult with a slightly higher, not statistically significant, dropout rate in the social compared with personal perspective (26% v. 20%). This difficulty may be attributed to several factors: the instructions were somewhat longer and less familiar, requiring participants to imagine the lives of other people and advise policy makers on behalf of a society, rather than stating their personal preferences. Consequently, respondents in the social preference survey reported greater uncertainty in their answers compared with the higher confidence expressed in the personal preference DCE. This increased cognitive load is further supported by the significantly longer median completion time for the social survey (11.3 min) compared with the personal survey (10.5 min). In addition, the social perspective exhibited a higher incidence of dominance violations in the practice tasks. Qualitative feedback also indicated that the social perspective garnered more negative comments and was perceived as more challenging, with 32 (1.1%) comments mentioning difficulty compared with 8 (0.8%) from personal survey respondents, highlighting the general challenge of weighing competing aspects, which was more pronounced in the social context. It may not come as a surprise that the social perspective is more difficult, but this study provides evidence to support this view.

However, it should also be noted that some respondents answering the personal DCE found this unrealistic because of their personal circumstances. For example, respondents who were retired reported that they found life situations involving full-time education or employment unlikely. Furthermore, the personal preference DCE comprised a smaller sample (*n* = 1,020) than the social preference DCE did (*n* = 3,009). Nonetheless, the extent of preference heterogeneity, as evidenced by the standard deviations in Supplementary Table 3, was not generally higher in the personal preference model relative to the social preference model. Consistent with our findings, a related study by Ta et al. (2024)^18^ reported similar personal preference patterns, notably for the employment attribute where being a carer was less preferred than all other employment categories.

One question for health economists is whether or not these results could be interpreted to mean that while perspectives may matter for the economic evaluation of public health and social care interventions, clinical interventions are not affected. It should be noted, however, that the elicitation exercise used in this study did not use the traditional QALY valuation framework, in which health state valuations ask respondents to trade off between full health and survival. The DCE exercise used in this study does not involve length or probability of survival as an attribute.

Furthermore, SIPHER-7 was developed through discussions with public health researchers and with UK local government policy actors responsible for public health.^15^ They were interested in well-being indicators used in large-scale social surveys, so that the well-being impacts of different public policy interventions can be modeled using secondary data. As a result, compared with well-being outcome measures in health economics such as ICECAP and EQ-HWB, SIPHER-7 has a broader coverage and includes indicators that lie beyond health. This makes it important to consider the possible interaction between the coverage of an outcome measure and the perspective used to value it. For example, trading off across more outward-facing items such as housing quality or employment may be better aligned to a social perspective, while trading off across more inward-facing items such as feeling secure or exhausted may be better aligned to a personal perspective. If so, our findings cannot be generalized to health and well-being measures that comprise items that are more inward facing. It would be interesting to conduct further comparisons across perspectives using different outcome measures.

The strengths of this study include using near-identical DCEs with a robust choice design to compare social and personal preferences with large sample sizes. The study also included 2 qualitative components: cognitive interviewing was used at the development stage to improve the understanding of tasks presented to the participants, and the free-text feedback was coded and compared across the perspectives to better understand the respondent’s reception of the survey.

On the other hand, there are some limitations. First, a considerable proportion of respondents failed a dominant practice task, suggesting, possibly, that the task is not suitable for some respondents to engage in through unfacilitated online surveys. However, when sensitivity analyses were conducted excluding these respondents, the results were still robust and no different from the main analysis.

Our findings suggest the perspective from which preferences are elicited can significantly affect valuations, particularly for attributes such as employment and housing quality. This underscores the need to consider which preferences are used when resource allocation decisions are made for public health and social care policies. Our work indicates that a personal perspective may be more accessible for participants. It also found that for attributes such as physical health, emotional problems, loneliness, and neighborhood safety, the 2 perspectives had no significant differences. These findings offer support for the use of a personal perspective for eliciting preferences in these domains. However, the perspective affected valuations significantly for employment and housing quality. Thus, researchers should carefully consider which perspective to use in future studies involving the more social domains of life, as it can directly influence the resulting valuations and subsequent policy recommendations.

## Supplemental Material

sj-docx-1-mdm-10.1177_0272989X251378427 – Supplemental material for Using Discrete Choice Experiments (DCEs) to Compare Social and Personal Preferences for Health and Well-Being Outcomes Supplemental material, sj-docx-1-mdm-10.1177_0272989X251378427 for Using Discrete Choice Experiments (DCEs) to Compare Social and Personal Preferences for Health and Well-Being Outcomes by Nyantara Wickramasekera, An Thu Ta, Becky Field and Aki Tsuchiya in Medical Decision Making
